# Correction: Recombinant protein KR95 as an alternative for serological diagnosis of human visceral leishmaniasis in the Americas

**DOI:** 10.1371/journal.pone.0293055

**Published:** 2023-10-12

**Authors:** The PLOS ONE Staff

There are errors in the Funding section. The correct Funding statement is: This study was supported by Fundação de Amparo à Pesquisa do Estado de São Paulo, grant number: 2021/12535-2 to MCAS (https://fapesp.br/) and Laboratório de Investigação Médica (LIM-38) Hospital das Clínicas da Faculdade de Medicina da Universidade de São Paulo (https://limhc.fm.usp.br/portal/). HG received a research fellowship, grant number: 3029440/2019-7, from Conselho Nacional de Desenvolvimento Científico e Tecnológico (https://www.gov.br/cnpq/pt-br). MF received a scholarship, grant number: 2017/03367-3, from Fundação de Amparo à Pesquisa do Estado de São Paulo (https://fapesp.br/). RTVP received a scholarship, grant numbers: 88882.376665/2019-01 and 88887.689454/2022-00, from Coordenação de Aperfeiçoamento de Pessoal de Nível Superior (https://www.gov.br/capes/pt-br).

The publisher apologizes for the error.

## References

[1] Fujimori M, Valencia-Portillo RT, Lindoso JAL, Celeste BJ, de Almeida RP, Costa CHN, et al. (2023) Recombinant protein KR95 as an alternative for serological diagnosis of human visceral leishmaniasis in the Americas. PLoS ONE 18(3): e0282483. 10.1371/journal.pone.0282483 36862710 PMC9980733

