# Supplementary material for: Recombinant protein KR95 as an alternative for serological diagnosis of human visceral leishmaniasis in the Americas
Source: PLoS One. 2023 Mar 2;18(3):e0282483. doi: 10.1371/journal.pone.0282483 (PMC9980733; doi:10.1371/journal.pone.0282483)
Supplement: S1 Table — n—number of samples; VL—visceral leishmaniasis; α—missing information of one VL patient; M—man; F—female; β—missing information of 16 VL patients; DAT—direct agglutination test; min-max—minimum-maximum; *—band test intensity; μ—21 samples from VL patients and one healthy control sample were not tested in the Kalazar Detect and IT-Leish tests. (DOCX) [file pone.0282483.s001.docx]

**S1 Table - Demographic and laboratory data for VL patients and healthy controls from endemic areas used to construct the ROC curves (Panel 1).**

| **Data** | | **VL patients**  **n=90** | **Healthy controls**  **n=90** |
| --- | --- | --- | --- |
| **Sex ^α^** | M | 63 | 46 |
|  | F | 26 | 44 |
| **Age (years) ^β^** | median | 21 | 23 |
|  | min-max | 1-86 | 1-90 |
| **DAT (titer)** | median | 102,400 | 100 |
|  | min-max | 100-204,800 | 100-100 |
| **Parasitology (myelogram)** | positive | 90 | not done |
| **Kalazar Detect* ^µ^** | median | 3 | 0 |
|  | min-max | 0-3 | 0-3 |
| **IT-Leish* ^µ^** | median | 3 | 0 |
|  | min-max | 0-3 | 0-3 |

n – number of samples; VL – visceral leishmaniasis; ^α^ – missing information of one VL patient; M – man; F – female; ^β^ – missing information of 16 VL patients; DAT – direct agglutination test; min-max – minimum-maximum; * – band test intensity; ^µ –^ 21 samples from VL patients and one healthy control sample were not tested by Kalazar Detect and IT-Leish tests.
