# Supplementary material for: Recombinant protein KR95 as an alternative for serological diagnosis of human visceral leishmaniasis in the Americas
Source: PLoS One. 2023 Mar 2;18(3):e0282483. doi: 10.1371/journal.pone.0282483 (PMC9980733; doi:10.1371/journal.pone.0282483)
Supplement: S2 Table — n—number of samples; VL—visceral leishmaniasis; DAT—direct agglutination test. (DOCX) [file pone.0282483.s002.docx]

**S2 Table – Collection date and diagnosis criteria for VL patients and healthy controls from endemic areas used in the ROC curves.**

| Panel 1 | Samples (n) | Collection date | Diagnosis |
| --- | --- | --- | --- |
| Campo Grande - Mato Grosso do Sul  (VL samples) | 42 | 2004 - 2008 | Infection was confirmed by the finding of *Leishmania* under microscopic examination in bone marrow aspirate and by positive DAT |
| Piaui  (VL samples) | 48 | 2008 - 2009 | Infection was confirmed by the finding of *Leishmania* under microscopic examination in bone marrow aspirate and by positive DAT |
| Tres Lagoas - Mato Grosso do Sul  (control samples) | 90 | 2003 | Negative by DAT and asymptomatic for VL for six months follow-up |

n – number of samples; VL – visceral leishmaniasis; DAT – direct agglutination test.
