# Supplementary material for: Recombinant protein KR95 as an alternative for serological diagnosis of human visceral leishmaniasis in the Americas
Source: PLoS One. 2023 Mar 2;18(3):e0282483. doi: 10.1371/journal.pone.0282483 (PMC9980733; doi:10.1371/journal.pone.0282483)
Supplement: S3 Table — n—number of samples; VL—visceral leishmaniasis; VL/AIDS—co-infection; α—missing information of three VL patients and 41 VL/AIDS patients; M—man; F—female; β—missing information of five VL patients and 41 VL/AIDS patients; min-max—minimum-maximum; DAT—direct agglutination test; *—band test intensity; μ—44 samples from VL/AIDS patients were not tested by DAT, Kalazar Detect and IT-Leish tests. (DOCX) [file pone.0282483.s003.docx]

**S3 Table - Demographic and laboratory data for VL patients, VL / AIDS patients, and healthy controls from various Brazilian endemic areas (Panel 2).**

| Data | | VL patients  n=122 | Healthy controls  n=83 | VL/AIDS patients n=64 |
| --- | --- | --- | --- | --- |
| **Sex ^α^** | M | 83 | 44 | 15 |
|  | F | 36 | 39 | 8 |
| **Age (years) ^β^** | median | 26 | 29 | 37 |
|  | min-max | 1-90 | 2-67 | 20 |
| **DAT (titer) ^µ^** | median | 102,400 | 100 | 25,600  100-409,600 |
|  | min-max | 200-409,600 | 100-1,600 |  |
| **Parasitology (myelogram)** | positive | 58 | 0 | 59 |
|  | negative | 20 | 6 | 3 |
|  | not done | 44 | 79 | 2 |
| **Kalazar Detect* ^µ^** | median | 3 | 0 | 1  0-3 |
|  | min-max | 0-3 | 0-1 |  |
| **IT-Leish* ^µ^** | median | 3 | 0 | 2  0-3 |
|  | min-max | 0-3 | 0-3 |  |

n – number of samples; VL – visceral leishmaniasis; VL/AIDS – co-infection; ^α^ – missing information of three VL patients and 41 VL/AIDS patients; M – man; F – female; ^β^ – missing information of five VL patients and 41 VL/AIDS patients; min-max – minimum-maximum; DAT – direct agglutination test; * – band test intensity; ^µ^ – 44 samples from VL/AIDS patients were not tested by DAT, Kalazar Detect and IT-Leish tests.
