# Supplementary material for: Recombinant protein KR95 as an alternative for serological diagnosis of human visceral leishmaniasis in the Americas
Source: PLoS One. 2023 Mar 2;18(3):e0282483. doi: 10.1371/journal.pone.0282483 (PMC9980733; doi:10.1371/journal.pone.0282483)
Supplement: S4 Table — n—number of samples; VL—visceral leishmaniasis; VL/AIDS—co-infection; DAT—direct agglutination test. (DOCX) [file pone.0282483.s004.docx]

**S4 Table –** **Collection date and diagnosis criteria for VL patients, VL /AIDS patients, and healthy controls from various Brazilian endemic areas (Panel 2).**

|  | City | n | Collection date | Diagnosis |
| --- | --- | --- | --- | --- |
| VL patients | 1 | 30 | 2014 - 2015 | Infection was confirmed by the finding of *Leishmania* under microscopic examination in bone marrow aspirate and/or by positive DAT |
|  | 2 | 50 | 2012 - 2014 |  |
|  | 3 | 27 | 2013 - 2014 |  |
|  | 6 | 15 | 2013 |  |
| VL / AIDS patients | 1 | 4 | 2014 - 2015 | Infection was confirmed by the finding of *Leishmania* under microscopic examination in bone marrow aspirate and/or by positive DAT  HIV infection was confirmed by the routine diagnostic methods of the center |
|  | 6 | 5 | 2013 |  |
|  | 3 | 11 | 2013-2014 |  |
|  | 3 | 32 | 2013-2014 | The samples were characterized using routine diagnostic methods of the center |
|  | 4 | 9 | 2008 |  |
|  | 5 | 3 | 2002 - 2003 |  |
| Healthy controls | 1 | 33 | 2014 - 2015 | Healthy individuals living in endemic areas and negative by DAT |
|  | 2 | 13 | 2012 - 2014 |  |
|  | 3 | 30 | 2013 - 2014 |  |
|  | 6 | 7 | 2013 |  |

n – number of samples; VL – visceral leishmaniasis; VL/AIDS – co-infection; DAT – direct agglutination test.
