# Supplementary material for: Recombinant protein KR95 as an alternative for serological diagnosis of human visceral leishmaniasis in the Americas
Source: PLoS One. 2023 Mar 2;18(3):e0282483. doi: 10.1371/journal.pone.0282483 (PMC9980733; doi:10.1371/journal.pone.0282483)
Supplement: S5 Table — n—number of samples. (DOCX) [file pone.0282483.s005.docx]

**S5 Table – Collection date and diagnosis criteria for potentially cross-reactive controls.**

| Panel 3 | Samples (n) | Collection date | Diagnosis |
| --- | --- | --- | --- |
| Autoimmune Disease | 10 | 2005 | IFI (indirect immunofluorescence) with HEp-2 cells/ ANA (Antinuclear Antibody) Test |
| Chagas Disease | 47 | 2006 - 2007/ 2014 | Positive serology ELISA/hemagglutination/ TESAcruzi |
| Cutaneous leishmaniasis | 28 | 2013 | Positive parasitological test |
| Malaria | 12 | 1986 - 2008 | Positive by thick-blood smear for *Plasmodium vivax* or *P. falciparum* |
| Mucosal leishmaniasis | 14 | 2013 - 2014 | Positive parasitological test |
| Paracoccidioidomycosis | 27 | 2006 | Positive by dual immunodiffusion and counter-immunoelectrophoretic assay |
| Syphilis | 20 | 2005 | Treponemal tests (FTA-Abs (fluorescent treponemal antibody absorption test)/ hemagglutination/ Western blotting) |
| Toxoplasmosis | 20 | 2005 | ELISA (IgG positive) with results of varied avidity |
| Tuberculosis | 12 | 2001 - 2002/ 2014 | Positive by bacilloscopic exam/*Mycobacterium tuberculosis* culture/biopsy |

n – number of samples.
