# Supplementary material for: Recombinant protein KR95 as an alternative for serological diagnosis of human visceral leishmaniasis in the Americas
Source: PLoS One. 2023 Mar 2;18(3):e0282483. doi: 10.1371/journal.pone.0282483 (PMC9980733; doi:10.1371/journal.pone.0282483)
Supplement: S6 Table — n—number of negative samples; 95% CI—95% probability confidence interval. Cochran’s Q test: a—p < 0.0001; b—p = 0.0027; c—p = 0.0006. Pairwise McNemar’s test: d—p = 0.0040—compared with rK28-ELISA and rKR95-ELISA; p = 0.0090—compared with rK39-ELISA. e—p = 0.0358—compared with rK39-ELISA and rKR95-ELISA; p = 0.0833—compared with rK28-ELISA. f—p = 0.0140—compared with rK28-ELISA and rKR95-ELISA. g—p = 0.0067—compared with rK28-ELISA, rK39-ELISA, and rKR95-ELISA. h—p < 0.0001—compared with rK28-ELISA, rK39-ELISA, and rKR95-ELISA. x—R cannot deal with factors containing only one level. (DOCX) [file pone.0282483.s006.docx]

**S6 Table. Specificity of rK18-ELISA, rK28-ELISA, rK39-ELISA, and rKR95-ELISA in sera from patients with other diseases (Panel 3).**

| **Diseases (n)** | **Specificity% (n) - 95% CI** | | | | | | | |
| --- | --- | --- | --- | --- | --- | --- | --- | --- |
|  | **rK28** | | **rK39** | | **rK18** | | **rKR95** | |
| **Autoimmune disease (10)** | 100.0 (10) | 67.9-100.0 | 100.0 (10) | 67.9-100.0 | 100.0 (10) | 67.9-100.0 | 100.0 (10) | 67.9-100.0 |
| **Chagas disease (47) ^a^** | 100.0 (47) | 90.9-100.0 | 100.0 (47) | 90.9-100.0 | 83.0 (39) ^x^ | 69.6-91.4 | 100.0 (47) | 90.9-100.0 |
| **Cutaneous leishmaniasis (28) ^a^** | 92.9 (26) | 76.3-99.1 | 89.3 (25) | 72.0-97.1 | 50.0 (14) ^d^ | 32.6-67.4 | 92.9 (26) | 76.3-99.1 |
| **Malaria (12) ^b^** | 66.7 (8) | 38.8-86.4 | 91.7 (11) | 62.5-99.9 | 41.7 (5) ^e^ | 19.3-68.1 | 91.7 (11) | 62.5-99.9 |
| **Mucosal leishmaniasis (14) ^c^** | 92.9 (13) | 66.5-99.9 | 78.6 (11) | 51.7-93.2 | 35.7 (5) ^f^ | 16.2-61.4 | 92.9 (13) | 66.5-99.9 |
| **Paracoccidioidomycosis (27) ^a^** | 96.3 (26) | 80.2-99.9 | 96.3 (26) | 80.2-99.9 | 63.0 (17) ^g^ | 40.7-75.5 | 96.3 (26) | 80.2-99.9 |
| **Syphilis (20) ^b^** | 95.0 (19) | 74.6-99.9 | 100.0 (20) | 81.0-100.0 | 70.0 (14) ^x^ | 47.9-85.7 | 100.0 (20) | 81.0-100.0 |
| **Toxoplasmosis (20)** | 100.0 (20) | 81.0-100.0 | 100.0 (20) | 81.0-100.0 | 95.0 (19) | 75.6-99.9 | 100.0 (20) | 81.0-100.0 |
| **Tuberculosis (12) ^a^** | 100.0 (12) | 71.8-100.0 | 100.0 (12) | 71.8-100.0 | 16.7 (2) ^x^ | 3.5-46.0 | 91.7 (11) | 62.5-99.9 |
| **TOTAL (190) ^a^** | 95.3 (181) | 91.1-97.6 | 95.8 (182) | 91.8-98.0 | 65.8 (125) ^h^ | 58.8-72.2 | 96.8 (184) | 93.1-98.7 |

n – number of samples; 95% CI – 95% probability confidence interval.

Cochran’s Q test:

a – p < 0.0001; b – p = 0.0027; c – p = 0.0006.

Pairwise McNemar’s test:

d – p = 0.0040 - compared with rK28-ELISA and rKR95-ELISA; p = 0.0090 compared with rK39-ELISA.

e – p = 0.0358 - compared with rK39-ELISA and rKR95-ELISA.

f – p = 0.0140 - compared with rK28-ELISA and rKR95-ELISA.

g – p = 0.0067 - compared with rK28-ELISA, rK39-ELISA, and rKR95-ELISA.

h – p < 0.0001 - compared with rK28-ELISA, rK39-ELISA, and rKR95-ELISA.

x – R cannot deal with factors containing only one level.
