# Supplementary figures and images for: Recombinant protein KR95 as an alternative for serological diagnosis of human visceral leishmaniasis in the Americas
Source: PLoS One. 2023 Mar 2;18(3):e0282483. doi: 10.1371/journal.pone.0282483 (PMC9980733; doi:10.1371/journal.pone.0282483)

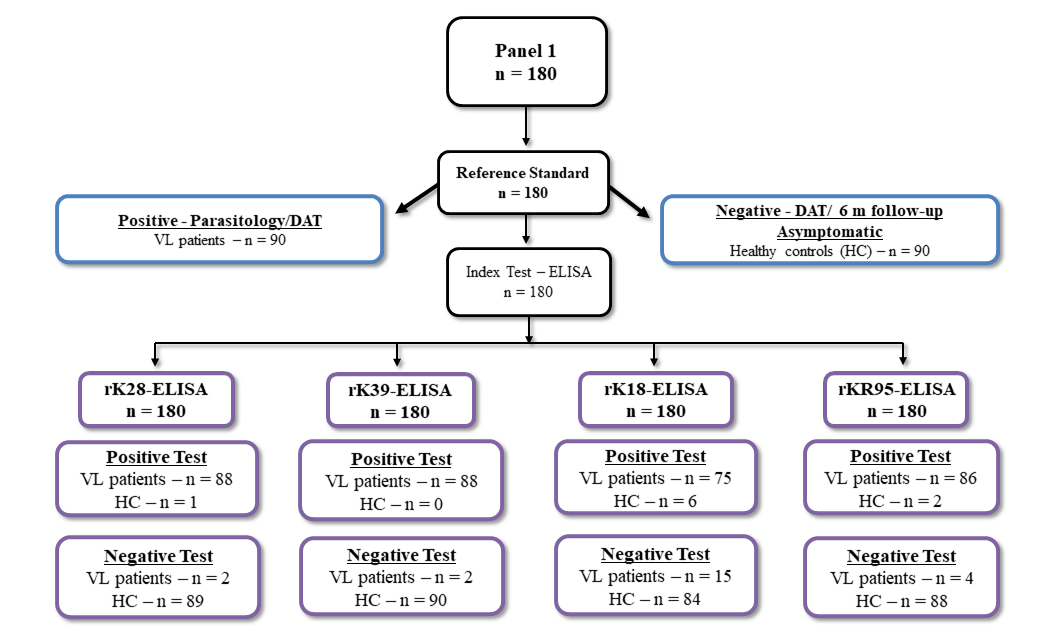

Supplement: S1 Fig — n—number of samples; VL—visceral leishmaniasis; DAT—direct agglutination test. (TIF) [file pone.0282483.s007.tif]

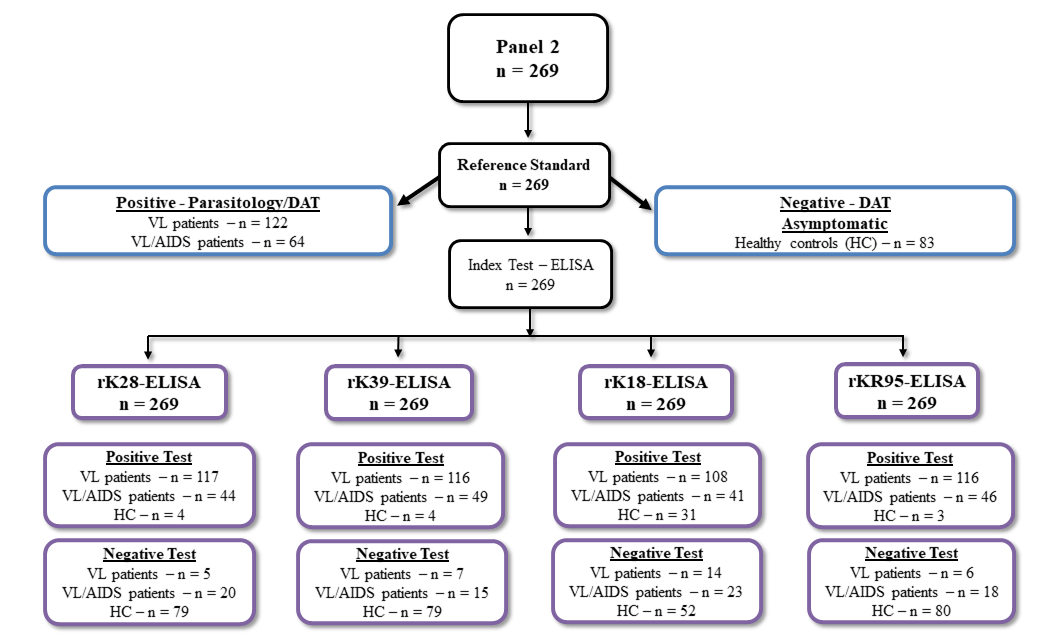

Supplement: S2 Fig — n—number of samples; VL—visceral leishmaniasis; VL/AIDS—co-infection; DAT—direct agglutination test. (TIF) [file pone.0282483.s008.tif]

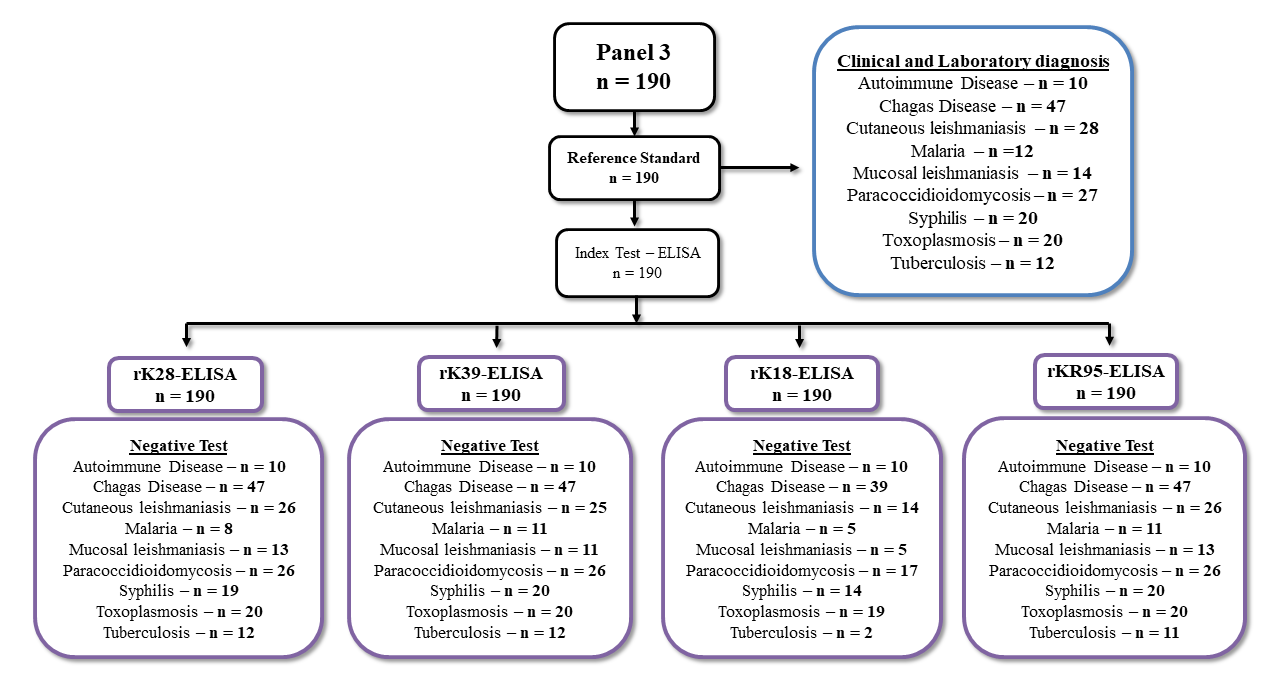

Supplement: S3 Fig — n—number of samples; VL—visceral leishmaniasis; VL/AIDS—co-infection; DAT—direct agglutination test. (TIF) [file pone.0282483.s009.tif]

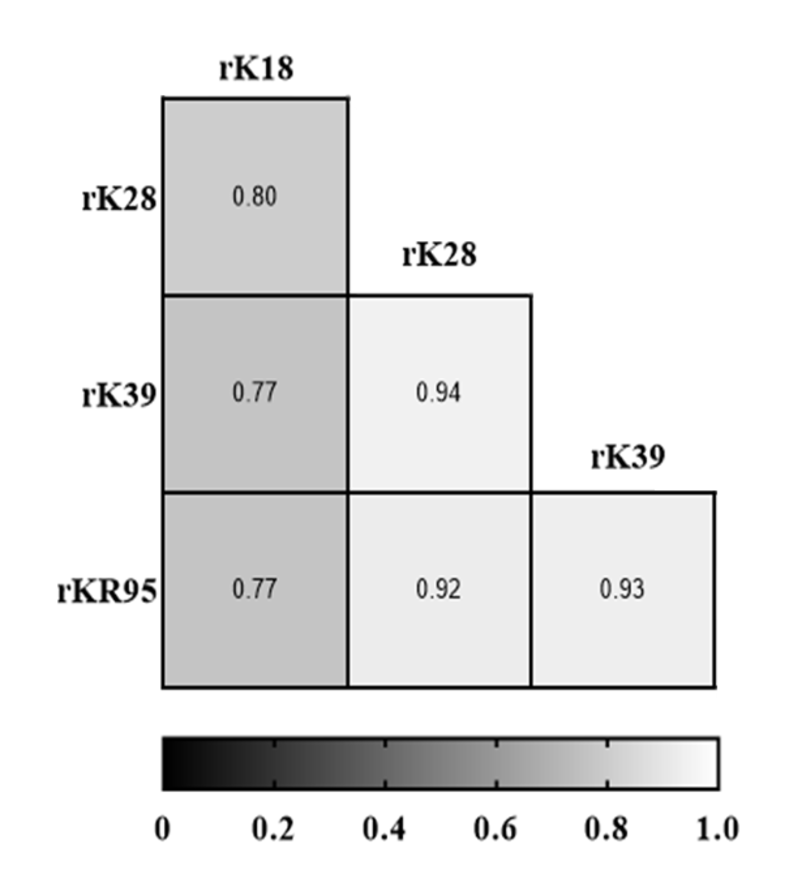

Supplement: S4 Fig — The heat map shows the correlation between the results obtained by rK28-ELISA, rK39-ELISA, rK18-ELISA, and rKR95-ELISA, two-by-two, testing samples of VL patients (n = 90) and healthy endemic controls (n = 90) (Panel 1). The strength of the correlation is displayed in colors ranging from black to white. (TIF) [file pone.0282483.s010.tif]

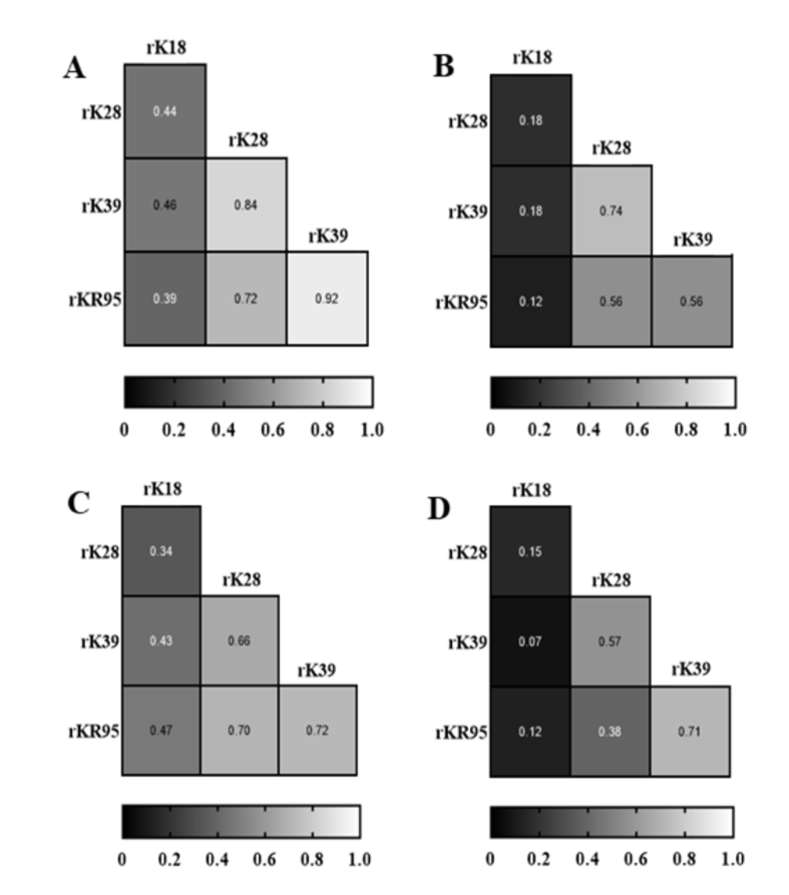

Supplement: S5 Fig — The heat map shows the correlation between the results obtained by rK18-ELISA, rK28-ELISA, rK39-ELISA, and rKR95-ELISA two-by-two. (A) One hundred twenty-two samples from VL patients, (B) 64 samples from VL / AIDS patients, (C) 83 samples from healthy controls, and (D) 190 samples from patients with other infectious diseases. The strength of the correlation is displayed in colors ranging from black to white. (TIF) [file pone.0282483.s011.tif]

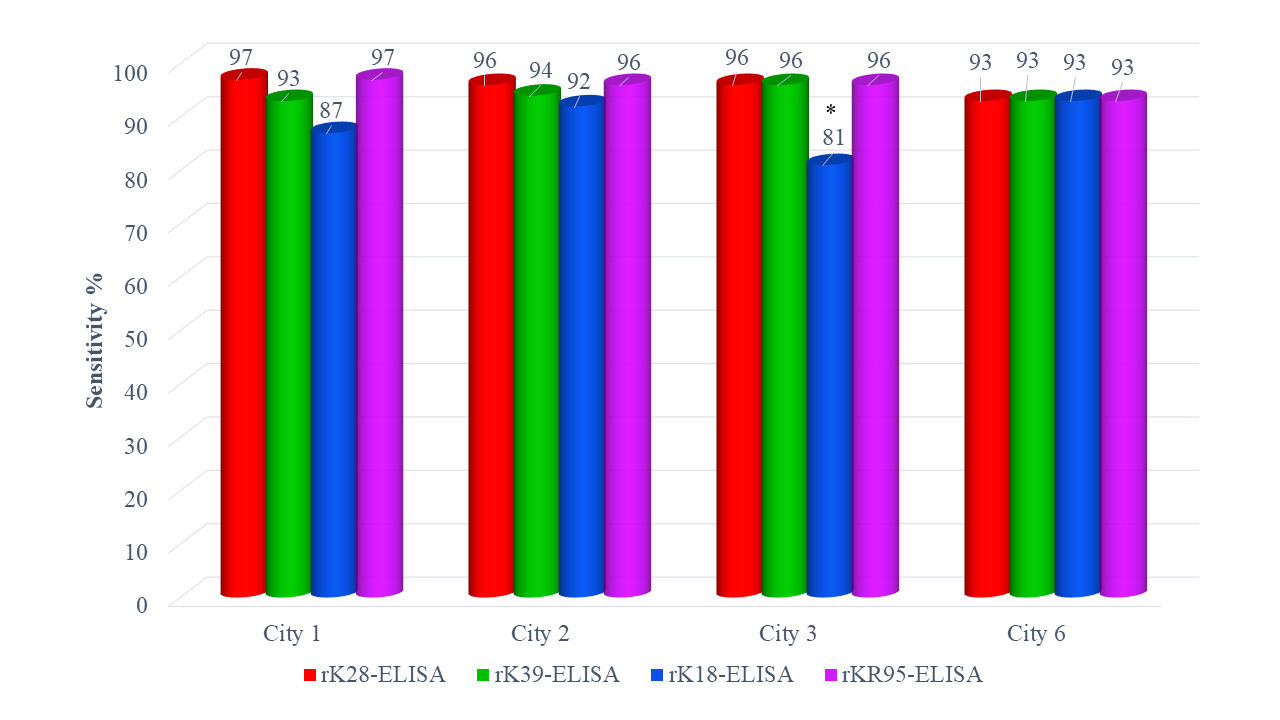

Supplement: S6 Fig — *—p = 0.0074 (Cochran’s Q test); p = 0.0455 (pairwise McNemar’s test) compared with rK28-ELISA, rK39-ELISA, and rKR95-ELISA. (TIF) [file pone.0282483.s012.tif]

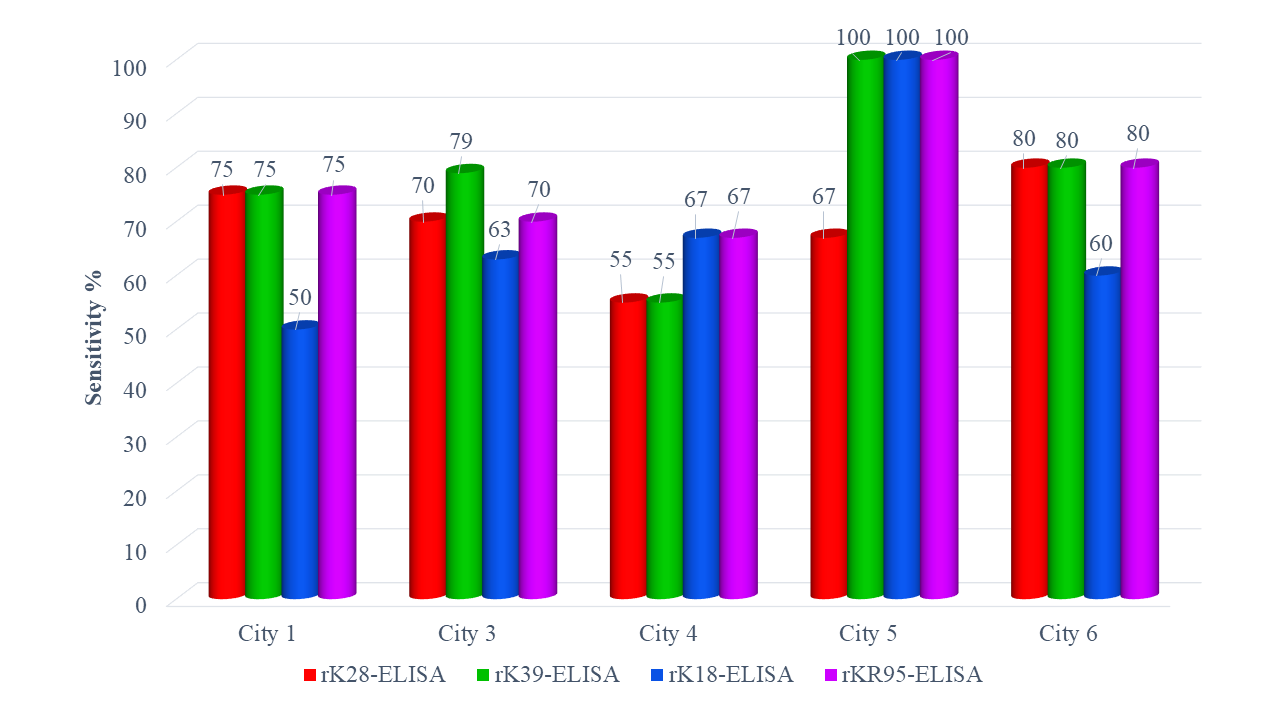

Supplement: S7 Fig — (TIF) [file pone.0282483.s013.tif]

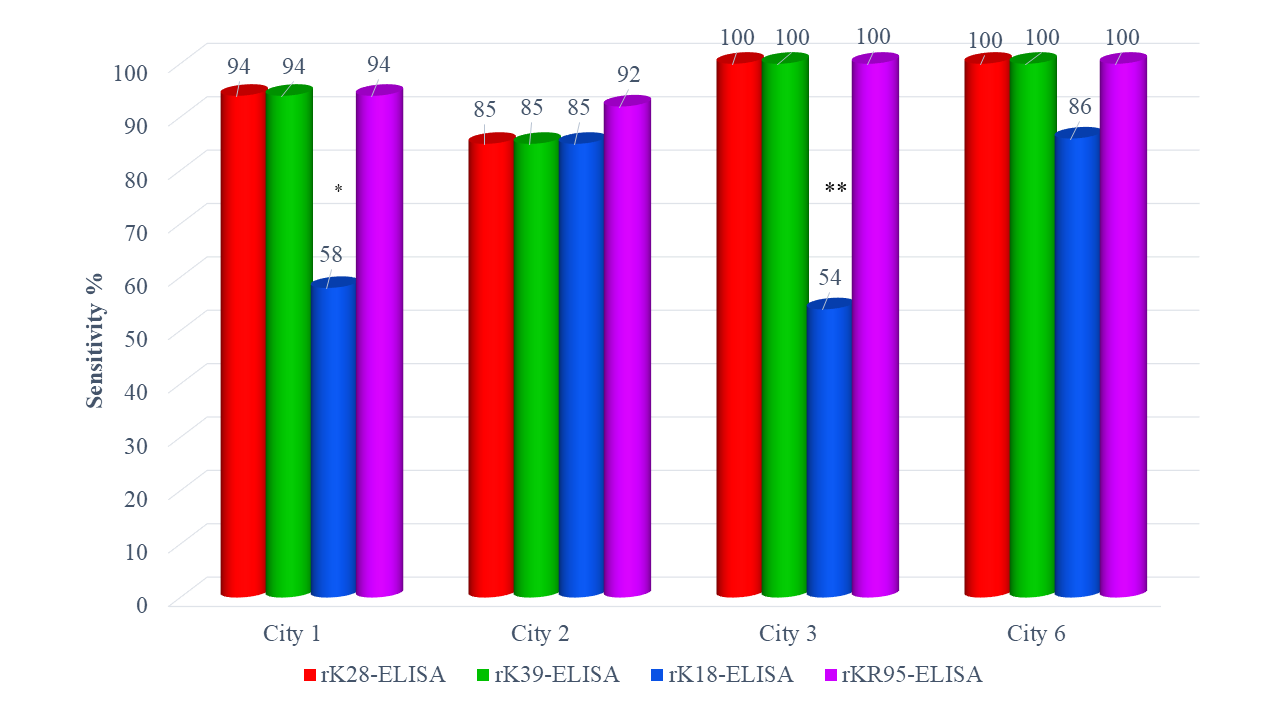

Supplement: S8 Fig — *—p < 0.0001 (Cochran’s Q test); p = 0.0002 (pairwise McNemar’s test) compared with rK28-ELISA, rK39-ELISA, and rKR95-ELISA. **—p < 0.0001 (Cochran’s Q test); p = 0.0016 (pairwise McNemar’s test) compared with rK28-ELISA and rK39-ELISA; p = 0.0027 (pairwise McNemar’s test) compared with rKR95-ELISA. (TIF) [file pone.0282483.s014.tif]
